# Supplementary material for: Association between Paraoxonase/Arylesterase Activity of Serum PON-1 Enzyme and Rheumatoid Arthritis: A Systematic Review and Meta-Analysis
Source: Antioxidants (Basel). 2022 Nov 23;11(12):2317. doi: 10.3390/antiox11122317 (PMC9774899; doi:10.3390/antiox11122317)
Supplement: Supplementary file 1 [file antioxidants-11-02317-s001.zip › Table S3.pdf]

Table S3: The Joanna Briggs Institute critical appraisal checklist.

Study	Were the criteria for inclusion clearly defined?	Were the subjects and the setting described in detail?	Was the exposure measured in a valid and reliable way?	Were objective, standard criteria used for measurement of the condition?	Were confounding factors identified?	Were strategies to deal with confounding factors stated?	Were the outcomes measured in a valid and reliable way?	Was appropriate statistical analysis used?	Risk of bias
Tanimoto N et al., 2003	No	No	Yes	Yes	Yes	No	Yes	No	Moderate
Baskol G et al., 2005	No	No	Yes	Yes	Yes	Yes	Yes	No	Low
Isik A et al., 2006	No	Yes	Yes	Yes	No	No	Yes	No	Moderate
Altindag O et al., 2007	No	Yes	Yes	Yes	No	No	Yes	No	Moderate
Charles-Schoeman C et al., 2012	No	Yes	Yes	Yes	Yes	Yes	Yes	Yes	Low
Sezer et al., 2013	Yes	Yes	Yes	Yes	Yes	Yes	Yes	Yes	Low
El-Banna H et al., 2014	Yes	Yes	Yes	Yes	Yes	Yes	Yes	No	Low
Shahmohamadnejad S., 2015	No	No	Yes	Yes	Yes	Yes	Yes	Yes	Low
Bindal UD et al., 2016	Yes	Yes	Yes	Yes	No	No	Yes	No	Low
Botta et al., 2016	Yes	Yes	Yes	Yes	Yes	Yes	Yes	Yes	Low
Keskin Y et al., 2016	No	Yes	Yes	Yes	No	No	Yes	No	Moderate
Rodriguez-Carrio et al., 2016	No	Yes	Yes	Yes	No	No	Yes	Yes	Low
O'Neill F et al., 2018	Yes	Yes	Yes	Yes	Yes	Yes	Yes	Yes	Low
Shevchuck SV et al., 2018	No	No	Yes	Yes	No	No	Yes	No	High
Yang L et al., 2018	No	Yes	Yes	Yes	No	No	Yes	No	Moderate
Mucientes A et al., 2019	No	Yes	Yes	Yes	No	No	Yes	No	Moderate
Atwa ET et al., 2020	Yes	Yes	Yes	Yes	No	No	Yes	Yes	Low
Bassu S et al., 2020	Yes	Yes	Yes	Yes	Yes	Yes	Yes	Yes	Low
Parada-Turska J et al., 2020	No	Yes	Yes	Yes	No	No	Yes	No	Low
Alisik T et al., 2021	No	Yes	Yes	Yes	No	No	Yes	No	Moderate
