# Supplementary material for: Association between Paraoxonase/Arylesterase Activity of Serum PON-1 Enzyme and Rheumatoid Arthritis: A Systematic Review and Meta-Analysis
Source: Antioxidants (Basel). 2022 Nov 23;11(12):2317. doi: 10.3390/antiox11122317 (PMC9774899; doi:10.3390/antiox11122317)
Supplement: Supplementary file 1 [file antioxidants-11-02317-s001.zip › Table S4.pdf]

Table S4. Certainty of evidence.

Outcome	Quality assessment					Quality
	Study design	Risk of bias	Inconsistency	Indirectness	Imprecision	
PON activity	Serious [a]<br>17 cross-sectional<br>Studies - 1144 participants	Not serious:<br>10 Low<br>6 Moderate<br>1 High	Serious[b]<br>I^2= 97%	No serious indirectness	Not serious<br>SMD -1.32<br>95%CI (-0.70 to -1.94)	⊕○○○<br>Very low
ARE activity	Serious [a]<br>10 cross-sectional<br>Studies - 1367 participants	Not serious:<br>6 Low<br>4 Moderate	Serious[b]<br>I^2= 95.7%	No serious indirectness	Not serious<br>SMD -0.91<br>95%CI (-0.91 to -0.46)	⊕○○○<br>Very low

[a]The certainty was downgraded of 2 levels due to the cross-sectional design of studies included in the meta-analysis

[b]The certainty of the evidence was downgraded of 1 level due to the high and unexplained heterogeneity
